# Problems of a Regimental Officer

**Published:** 1918-09

**Authors:** 


**Problems of a Regimental Officer**. Major Cowie, R. A. M. C.

The speaker said he had spent most of his life as a regimental officer, and, as such, he had come to realize that this war is a bloody business. He wanted Americans to know this. It had been a great pleasure to him to observe the cordial relations between the British and Americans in general, and he was much pleased with the hearty reception given to the British representatives at the Conference.

With regard to wastage, he believed there would be less wastage if there were more prevention; if, secondly, the doubtful cases could be kept with the regiment and the others brought back quickly to their unit; and if, thirdly, the permanency of the regimental medical officer could be assured.

In the matter of sanitation, the speaker believed the most important thing to be the education of the army officers, so that they might feel it to be the duty of each individual officer, and not merely that of the regimental doctor to maintain the health of the regiment.

There are engineering schools, artillery schools. and why should there not also be sanitation schools where army officers might be taught sanitation? These should be conducted by staff officers and not by medical officers.

The speaker said that he hoped he might live to see the time when there would be lively competition to enter the regimental medical service. The regimental M. O., as a young man, has at present nothing to look forward to. People were often heard to say that the regimental M. O. was a nuisance, and that if he " had any guts " he would have left the regimental office long ago. Major Cowie believed that these officers should be given the same chances of promotion as other medical corps men.

It was highly desirable to keep men with slight diseases in touch with their regiment, and proper provision should be made to accommodate all such without the necessity of sending them down to the hospital. In this way almost all light affections, like skin, throat, and digestive disturbances, might be made less wasteful to the army.

For this purpose every regiment should be equipped with two Bell tents for isolating cases. A stove should also be provided in every regiment to aid in the treatment of cases requiring warmth. The Primus stove is excellent for the purpose.

